# Colorectal cancer metastases in thyroid: case report and literature review

**DOI:** 10.1186/s13044-023-00150-y

**Published:** 2023-04-10

**Authors:** Syed Mohammad Asim Hussain, Suzanne Cole, Iram Hussain

**Affiliations:** 1 Vascular Surgery Department, Kent and Canterbury Hospital, East Kent Hospitals University, NHS Foundation Trust, Ethelbert Road, Canterbury, CT1 3NG UK; 2 Division of Oncology, Department of Internal Medicine, University Hospital Simmons Comprehensive Cancer Center at University of Texas Southwestern Medical Center at Richardson/Plano, 3030 Waterview Parkway, Richardson, TX 75080 USA; 3 Division of Endocrinology, Department of Internal Medicine, University of Texas Southwestern Medical Center, 5323 Harry Hines Boulevard, Dallas, TX 75390-8537 USA

**Keywords:** Colorectal cancer, Thyroid metastases, FNA biopsy, Fine needle aspiration, Thyroid nodule, Thyroidectomy, Immune markers, Cytology, Non-thyroid malignancy

## Abstract

**Background:**

The thyroid gland is an uncommon site for metastatic deposits from non-thyroid malignancies, occurring in only 1.4 - 3% of surgical specimens where malignancy is suspected. It is even rarer for the source of thyroid metastases to be of colorectal origin. In most cases reported, colorectal metastases in the thyroid occurs many years later after the primary colorectal cancer has been diagnosed and treated. In this unique case, a primary sigmoid carcinoma metastasised to the thyroid gland and presented synchronously as a thyroid nodule.

**Case presentation:**

We describe a case of a 64-year-old Caucasian woman who presented with clinical features of metastatic cancer of unknown origin. Her medical history included underlying hyperthyroidism. She had a large pelvic mass adjacent to the sigmoid colon, a left lower lobe lung mass and a suspicious nodule in the left thyroid lobe. A fine-needle aspiration biopsy of the thyroid nodule was performed, which remarkably showed malignant cells originating from primary colorectal cancer on immunohistochemical staining. The patient was managed with palliative chemotherapy given the poor prognosis due to disseminated colorectal malignancy.

**Conclusions:**

Colorectal adenocarcinoma metastases can rarely present as a metastatic thyroid nodule. Fine-needle aspiration should be performed in suspicious thyroid nodules and may be the least invasive way of identifying a metastatic colorectal or other non-thyroidal malignancy in patients presenting with an unknown primary. The pathologist should be vigilant to this possibility and specific immunohistochemical markers should be used to ensure accurate diagnosis.

In thyroid metastases, the prognosis is ultimately determined by the primary tumour but thyroidectomy still has a role in alleviating compressive symptoms and can potentially improve survival in selected cases.

## Background

The thyroid gland is an uncommon site for metastatic disease. It is estimated that metastases from non-thyroid malignancies account for only 1.4 to 3% of cases undergoing surgery for suspected thyroid cancer [1]. Out of these, renal carcinoma is the most common non-thyroid malignancy to metastasise to the thyroid gland in clinical studies [1].

A literature search was conducted to identify articles describing colorectal cancer metastasising to the thyroid gland in multiple databases, including PubMed, Cochrane Library and MEDLINE. The reference lists of identified articles were also reviewed for additional relevant articles. Colorectal cancer metastasising to the thyroid gland is an extremely rare phenomenon. Lievre et al. observed only 6 (0.1%) such cases amongst 5862 patients with colorectal cancer over an 11-year period [2]. A systematic review published in 2013 found only 34 cases of colorectal metastases in the thyroid since 1931 [3]. Among them, just five cases reported synchronous diagnosis of colorectal cancer and thyroid metastases.

Since the review in 2013, there have been at least 14 cases describing thyroid metastases of colorectal origin, which are outlined in Table 1. No case was diagnosed synchronously. There were 10 females and 4 males, and the mean age was 61 years. One patient reported by Mennet et al. had a multinodular goitre and another patient reported by Hernandez et al. had sub-clinical hypothyroidism, whereas the others did not have associated thyroid pathology [4, 5]. Four patients were incorrectly suspected of having papillary thyroid cancer on cytologic evaluation of fine needle aspiration (FNA) sample. All patients underwent thyroid surgery and were confirmed to have colorectal carcinoma metastases on histopathology.

**Table 1:** Case reports describing colorectal cancer metastases in thyroid gland since 2013

Author, Year	Age (y), Sex (M/F)	Cancer Origin	Primary Cancer TX	Time (y)	Studies	Thyroid Met TX	Other Metastatic Sites
Ozawa et al., 2015 [6]	72, F	Rectum	Anterior Resection	4	US, FNA, PET	ST	Lung, Adrenal
Roloff et al., 2016 [7]	42, F	Caecum	R hemicolectomy, adjuvant chemotherapy	2	US, FNA	TT	Liver, Lung
Mennet et al., 2016 [4]	68, M	Sigmoid	Sigmoid colectomy, adjuvant chemotherapy	2	PET, FNA – negative	L PT, TT & LND	Iliac-parietal nodules, Recto-vesical pouch
Minami et al., 2016 [8]	61, F	Sigmoid	Sigmoid colectomy	5	US, FNA -?PTC	TT & LND	Lung, Liver
Nicosia et al., 2016 [9]	75, M	Sigmoid	Sigmoid colectomy	6	PET, US, FNA	R PT	Lung
Coelho et al., 2017 [10]	64, F	Sigmoid	Sigmoid Colectomy, adjuvant chemotherapy	6	PET, US, FNA	R PT & LND	Lung, Liver, Pleura, Pericardium, Peritoneum
Dong-il et al., 2018 [11]	50, M	Descending colon	L hemicolectomy, adjuvant chemotherapy	4	PET	R PT & selective LND	Para-aortic nodes, Cervical nodes
Scepanovic et al., 2020 [12]	71, M	Rectum	Abdominoperineal resection, adjuvant chemotherapy	4	CT neck, FNA	TT	Lung
Zafar et al., 2020 [13]	57, F	Ascending Colon	R hemicolectomy, adjuvant chemotherapy	5	PET, US, FNA -? PTC	TT & LND	Lung
Mori et al., 2020 [14]	45, F	Rectum	Low Anterior Resection	5	CT, PET, US, FNA -Non-Diag	R PT & LND	Lung
Rojo-Abecia et al., 2020 [15]	85, F	Caecum	R hemicolectomy	14	PET, FNA	R PT	Lung
Hernandez et al., 2020 [5]	50, F	Rectum	Neo-adjuvant chemo-radiotherapy, anterior resection & total mesorectal excision	3.5	US, CT, FNA -? PTC	TT & LND	Lung
Momin et al., 2022 [16]	62, F	Caecum	R hemicolectomy	6	US, FNA, MRI neck, PET	R PT	Lung Liver
Mardani et al., 2022 [17]	55, F	Descending colon	L hemicolectomy, adjuvant chemo-radiotherapy	8	PET, FNA -? PTC	TT & LND	Lung

*Abbreviations*: *Time* The number of years to develop thyroid metastases after initial diagnosis of colorectal cancer, *y* Years, *Studies* Investigations and diagnostic studies done to evaluate thyroid metastases, *Met* Metastases, *TX* Treatment/management; *US* Ultrasonography, *FNA* Fine needle aspiration, *PET* Positron emission tomography, *PTC* Papillary thyroid carcinoma, *CT* Computed tomography, *MRI* Magnetic resonance imaging, *ST* Subtotal thyroidectomy, *TT* Total thyroidectomy, *L* Left, *R* Right, *PT* Partial thyroidectomy, *&* And, *LND* Lymph node dissection, *Non-Diag* Non-diagnostic,? Questionable

We present a case of metastatic colorectal cancer in the thyroid gland, diagnosed by FNA biopsy prior to histopathological confirmation of the primary colonic lesion.

## Case presentation

A 64-year-old woman presented to the Oncology department with a 6-month history of lower abdominal pain associated with blood-tinged mucus per rectum and unintentional weight loss. She also had progressive shortness of breath, coughing fits associated with haemoptysis, left sided chest pain radiating to the back and intermittent dysphagia with globus sensation. The patient reported a palpable pea-size nodule that appeared about 4 weeks prior to meeting with her primary care physician. Over a one-month time period the thyroid nodule rapidly increased to the size of a walnut.

On examination, she had a lump in the neck associated with cervical lymphadenopathy. Her neck mass became more prominent and moved with deglutition consistent with a thyroid nodule.

Her previous history included 3 caeserian sections with no prior history of malignancy. She was an ex-smoker. Her medications included acetaminophen for pain. There was a family history of breast cancer and Parkinson’s disease.

Laboratory evaluation revealed hyperthyroidism with undetectable thyroid stimulating hormone (TSH) and mildly elevated free thyroxine (FT4), for which she was started on methimazole 5 mg daily. She was subsequently noted to have elevated thyroid stimulating immunoglobulin and thyroglobulin antibody levels. She also had low haemoglobin, low mean corpuscular volume (MCV), low iron saturation and high total iron-binding capacity indicating iron-deficiency anaemia. The liver function tests were mildly deranged, and hyperglycaemia and hypercalcaemia were also observed. Mammography did not reveal evidence of a breast malignancy.

**Fig. 1:**
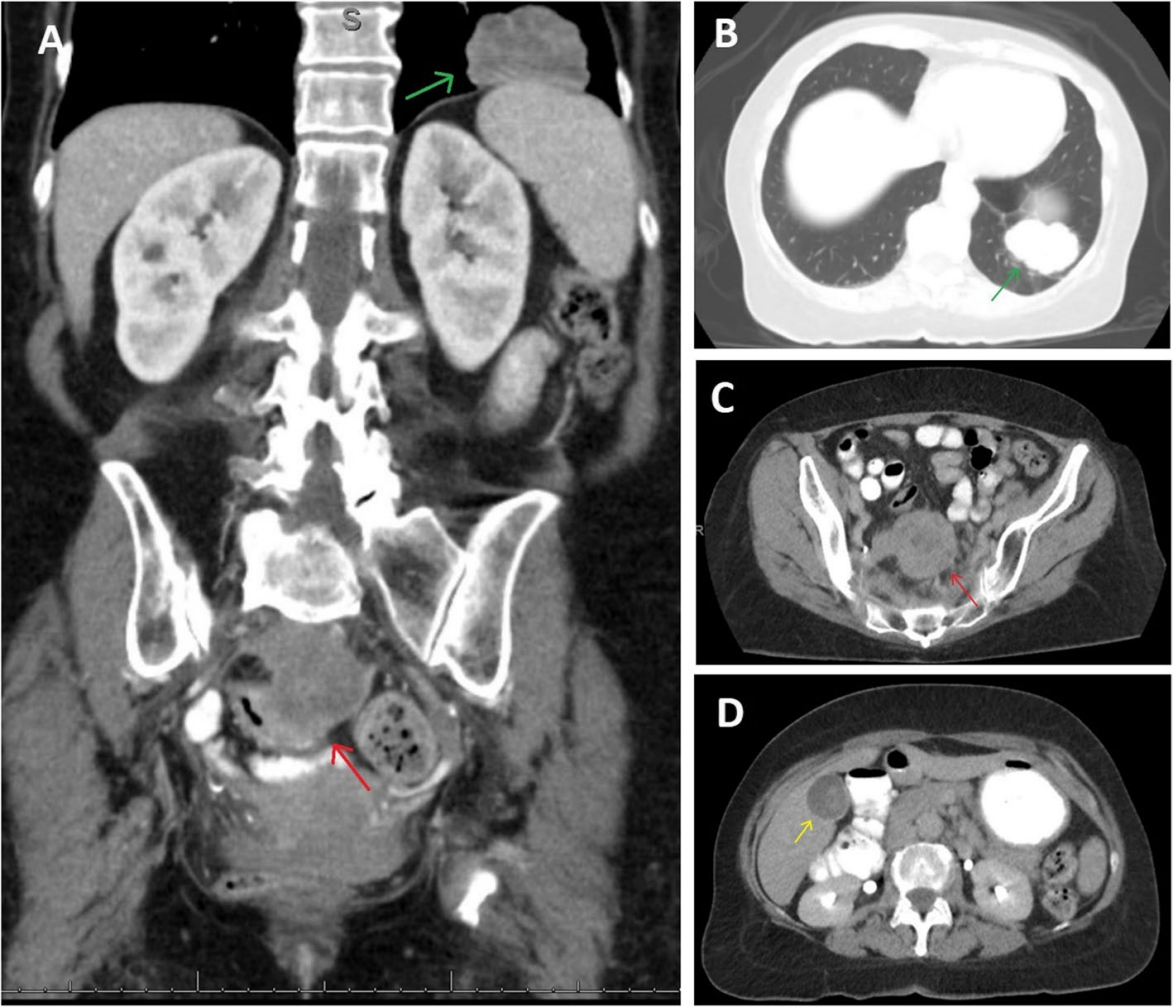
Computed tomography (CT) of abdomen and pelvis. This shows a CT image of the patient’s abdomen, and pelvis, along with the lower lung fields. **A** Coronal view of CT abdomen and pelvis with red arrow pointing to a large, midline mass at the lower retroperitoneal/upper pelvis immediately adjacent to the sigmoid colon measuring 5.8 cm X 5.4 cm X 5 cm consistent with sigmoid adenocarcinoma, and green arrow pointing toward left lower lung mass measuring 4.6 cm X 3.4 cm consistent with pulmonary metastasis. **B** Axial view with green arrow pointing towards the pulmonary metastases in the left lower lung. **C** Axial view with red arrow pointing towards the primary sigmoid adenocarcinoma. **D** Axial view with yellow arrow pointing to hypodense lesion in the liver consistent with liver metastasis

Computed Tomography (CT) of the abdomen and pelvis with and without contrast initially performed at an outside facility because of her abdominal pain revealed a left lower lobe lung mass measuring 4.6 cm X 3.4 cm (Fig. 1A and B) and pelvic mass immediately adjacent to the sigmoid colon measuring 5.8 cm X 5.4 cm X 5 cm (Fig. 1A and C). There were also multiple, hypodense lesions seen in the liver (Fig. 1D).

A thyroid ultrasound on the same day showed a solid 3.8 cm nodule in the left lower pole of the thyroid, which was taller than wide, hypoechoic and had irregular margins. This was classified as a moderately suspicious nodule requiring FNA biopsy per the American College of Radiology Thyroid Imaging Reporting and Data System (ACR-TIRADS).

The patient underwent a work-up for cancer of unknown primary. Given how widespread the disease was, and the patient reported rapid growth of the nodule, there was concern that the primary tumour could be a highly aggressive malignancy such as anaplastic thyroid cancer. The other possibility was a follicular thyroid cancer which can be associated with hyperthyroidism.

**Fig. 2:**
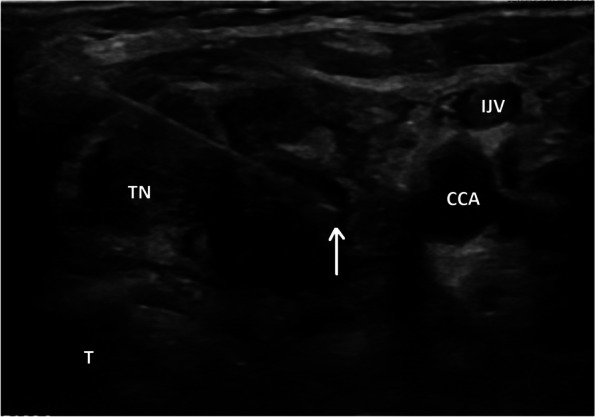
Ultrasound guided fine needle aspiration (FNA) of suspicious thyroid nodule. Ultrasound images showing 3.8 cm, hypoechoic thyroid nodule (TN) with irregular margins. The entire length of the needle inserted into the thyroid nodule during the FNA biopsy can be seen; the tip of the needle is indicated by the white arrow. T: trachea, CCA: common carotid artery, IJV: internal jugular vein

**Fig. 3:**
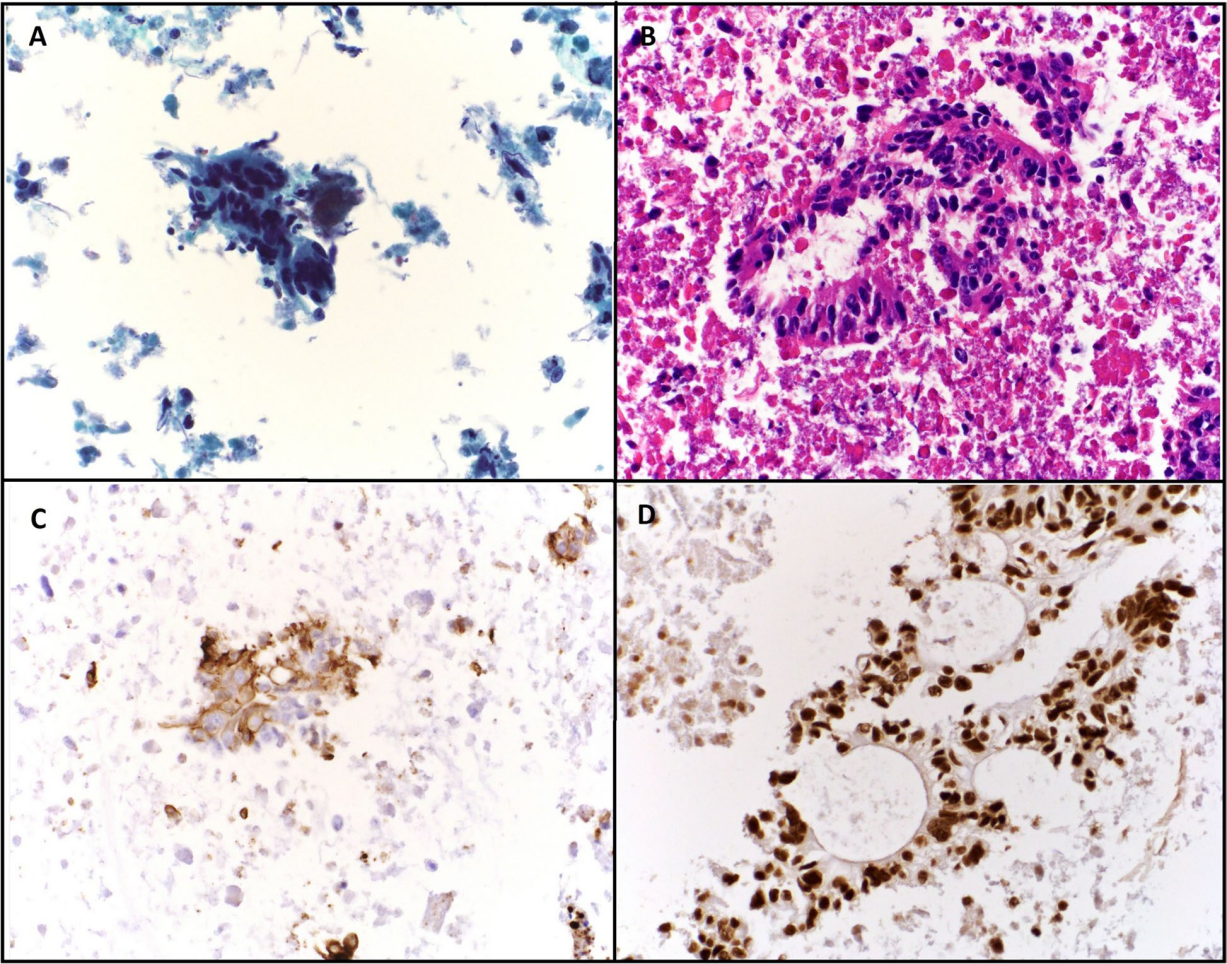
Cytopathology and immunohistochemical staining of fine needle aspiration (FNA) sample from thyroid nodule. Cytopathology showed malignant features consistent with a metastatic adenocarcinoma of gastrointestinal origin, favouring a colorectal primary. Magnification is 400x. **A** ThinPrep with Papanicolaou stain shows clusters of malignant columnar cells forming glands with nuclear pleomorphism and hyperchromasia. Background “dirty necrosis” is evident. **B** Hematoxylin and eosin stained cell block sections show malignant columnar cells in glandular formations and background necrosis. **C** Immunohistochemical stain for CK-20 shows positive cytoplasmic staining. **D** Immunohistochemical stain for CDX-2 shows positive nuclear staining

An FNA biopsy of the thyroid nodule was performed (Fig. 2). The specimen contained abundant cellularity and showed crowded aggregates of pleomorphic cells with hyperchromasia on a background of abundant necrosis (Fig. 3A and B). The pathologist noted that although the cells looked malignant, they did not appear to be of thyroid origin so additional immunohistochemical staining was performed.

Immunohistochemical staining showed the tumour cells were diffusely positive for cytokeratin 20 (CK20) (Fig. 3C) and caudal-related homeobox transcription factor 2 (CDX2) (Fig. 3D) while cytokeratin 7 (CK7), Wilms tumour 1 (WT1) and tumour protein 63 (p63) were all negative. Thyroid transcription factor 1 (TTF-1) and paired box protein (Pax8) were negative in tumour cells but showed very rare positivity in rare background thyroid follicular cells.

The final pathology findings showed malignant features consistent with metastatic adenocarcinoma of lower gastrointestinal tract origin with primary colorectal malignancy favoured (Fig. 3).

An ultrasound guided core biopsy of the 3 cm X 4 cm lesion in the right lobe of the liver was subsequently performed and supported the diagnosis of metastatic colorectal carcinoma with similar immune markers. Her plasma tumour markers showed elevated carcinoembryonic antigen (CEA) and lactate dehydrogenase (LDH) which provided further evidence for a colorectal malignancy.

As the patient was in significant pain, urgent chemotherapy treatment with 5-fluorouracil and oxaliplatin was commenced along with supportive medications including antiemetics, painkillers, prochlorperazine and dexamethasone. Given the widespread dissemination of malignancy, chemotherapy was initiated with palliative intent. She underwent 3 cycles after which she presented with a perforation at the site of the sigmoid colon tumour and subsequently underwent a diverting loop transverse colostomy.

## Discussion

Our case is novel because of the unusual presentation of colorectal cancer as a neck mass associated with cervical lymphadenopathy, along with the concurrent diagnosis of autoimmune hyperthyroidism. The diagnosis was made with ultrasound imaging and FNA biopsy of a symptomatic nodule. This is in contrast with other cases in which thyroid metastases were diagnosed incidentally on positron emission tomography (PET) or CT imaging; or were confirmed only after thyroidectomy on histopathology.

The synchronous diagnosis of colorectal metastases in the thyroid gland with the primary colorectal malignancy is rare. Only 5 out of 34 (14.7%) such cases were reported in a systematic review of thyroid metastases originating from primary colorectal cancers [3]. In our own review of 14 published cases since 2013, none of the colorectal thyroid metastases were diagnosed synchronously; rather the metastatic deposits in the thyroid gland were observed years after the initial diagnosis and surgical treatment of the colorectal carcinoma.

The timescale for detecting thyroid metastases following colonic resection ranged from 2 years to 14 years with a mean of 5.3 years. Our patient was symptomatic for only 5 months before presentation highlighting the rapid rate at which untreated colorectal cancer can disseminate. This suggests that cancer resection and chemo-radiotherapy may have primarily delayed metastatic spread to the thyroid in the cases reviewed.

In Froylich’s review, an associated thyroid pathology was found in 11 out of 34 (32.4%) cases of thyroid metastases suggesting some role for intrinsic thyroid characteristics in reducing metastatic deposits [3]. This finding was not replicated in our review since 2013, where only 2 out of 14 (14.2%) cases had an associated thyroid pathology; benign multinodular goitre in one case and subclinical hypothyroidism in another. In 1931, it was hypothesised that fast arterial blood flow in the thyroid prevents adhesion of cancer cells, and the high oxygen saturation and iodine content inhibits malignant cells [18]. Since then, there is increasing evidence of the anti-neoplastic effects of iodine on several cancer cell lines [19].

Our patient had hyperthyroidism which is associated with increased intra-thyroid blood flow and blood velocity [20]. Hyperthyroid states also decrease the half-life of iodine [21], which is consumed more rapidly to produce thyroid hormones resulting in decreased thyroid iodine content. It is possible that the increased thyroid blood flow and decreased thyroid iodine content increased the propensity for metastatic deposits in our patient. Of note, hyperthyroidism associated with infiltration of the thyroid gland by a non-thyroid malignancy is typically due to thyroiditis [22]. To our knowledge only one case of hyperthyroidism associated with colorectal carcinoma metastases to the thyroid has been reported, and this patient had negative thyroid antibodies [23]. Our patient is unique in that she has positive thyroid antibodies consistent with autoimmune thyroid disease and was treated with anti-thyroid medication.

The anatomical distance between the sigmoid colon and the thyroid gland is noteworthy in considering metastatic spread. Given that our patient also had liver and lung metastases, the most likely route was hematogenous spread via the portal vein to the liver. From there, it likely disseminated into the inferior vena cava, the right heart and pulmonary circulation before mostly depositing in the lung. Any cancer cells not deposited in the liver or lung likely got pumped out of the heart before seeding in the thyroid. Our review shows that the lungs were a metastatic site in 12 out of 14 (86%) cases. Interestingly, almost all cases of lung metastases underwent lung resection and chemotherapy. Surgical manipulation and cytoreductive therapy can cause embolism of cancer cells [24], which could enter the systemic circulation and deposit in the thyroid gland via the superior and inferior thyroid arteries.

The liver was a metastatic site in only 3 out of 14 (21.4%) of cases reviewed. This is unusual as generally the liver is the most common site for colorectal metastases [25]. An alternative metastatic pathway involves dissemination of rectal cancer via the middle and inferior rectal veins which drain into the iliac veins and inferior vena cava, bypassing the portal circulation to the liver. This pathway can explain incidences of colorectal cancer resulting in lung metastases without liver metastases.

We note that 4 out of 14 (28.6%) cases originated from the rectum and all of them spread to the lung. The caecum was a malignant source in 3 out of 14 (21.4%) cases, two of which spread to the liver. The sigmoid colon was a metastatic source in 4 out of 14 (28.6%) cases, two of which spread to both liver and lung, one metastasised to only the lungs and the remaining spread to the iliac-parietal nodules and recto-vesical pouch. The descending colon was a neoplastic source in 2 out of 14 (14%) and the ascending colon was an originating site in 1 out of 14 case (7.1%).

Colorectal cancer also commonly spreads through the lymphatic system to the regional lymph nodes. However, lymphatic spread is unlikely to account for thyroid metastases of colorectal origin as lymphatic flow is generally antegrade and unidirectional towards the subclavian veins [26]. The cervical lymphadenopathy seen in this case is likely the effect of the presence of metastatic cancer cells already within the thyroid, which originated hematogenously, and it is likely not the source of the thyroid metastatic cancer cells.

The clinical presentation in this case was of an aggressive malignancy. The patient reported typical symptoms of colorectal cancer including abdominal pain, rectal bleeding, and she did present with iron-deficiency anaemia. She also described symptoms suggesting lung cancer such as left sided radiating chest pain, shortness of breath and coughing fits with haemoptysis. Finally, the presence of a neck lump and cervical lymphadenopathy in association with a history of dysphagia invited consideration of underlying thyroid cancer. This differential diagnosis was supported by the imaging findings of a large retroperitoneal mass adjacent to the sigmoid colon, significant lower lobe lung mass and a sizeable left lobe thyroid nodule.

A histological diagnosis was important to determine treatment strategies and prognosis. The options included colonoscopy with colorectal biopsy, CT guided lung biopsy, ultrasound guided liver biopsy or ultrasound guided FNA biopsy of the thyroid gland. We proceeded with FNA of the thyroid first given the relative ease, efficiency and convenience of a thyroid biopsy which can be performed quickly in a clinic room with or without local anaesthetic with minimal patient discomfort.

The main investigations used to diagnose thyroid metastases are PET, CT, ultrasonography and FNA [27]. Interestingly, cases of colorectal thyroid metastases are reported more frequently from 2013 onwards compared to published cases from 1931 until 2013. We suggest that an increase in investigations and improvement in diagnostic techniques such as PET, CT and FNA is the explanation for this. Colorectal thyroid metastases are simply being diagnosed more often now and the actual incidence is unchanged and not as rare as once thought.

The ultrasonographic features indicating thyroid malignancy include hypo-echogenicity, microcalcifications, irregular margins, taller-than-wide appearance and increased intra-nodular vascularisation. FNA was indicated in our patient as the nodule was of a large size and demonstrated several malignant features presenting an estimated risk of malignancy of 70 – 90% as per the American Thyroid Association 2015 thyroid nodule and cancer guidelines and 5 – 20% per the ACR-TIRADS guidelines [27, 28]. There are no classification systems specifically for thyroid nodules that may be metastatic in origin.

FNA can accurately detect papillary, medullary, and anaplastic thyroid cancers and the literature suggests a sensitivity of 89% and specificity of 92% for primary thyroid cancer excluding follicular types [29]. It’s accuracy for the detection of metastatic thyroid cancer is less clear with 73.7% of cases correctly diagnosed and 24% of cases incorrectly identified in one systematic review [1]. The diagnostic accuracy is affected by the type of malignancy. Metastatic adenocarcinomas of breast, lung and colorectal origin were often correctly diagnosed whereas metastatic squamous cell carcinomas of oesophageal and cervical origin proved difficult to diagnose accurately [1]. FNA accurately diagnosed colorectal cancer thyroid metastases in our case.

In our review, PET scanning primarily identified suspicious thyroid nodules in 8 cases, ultrasound was primarily used in 5 cases and CT was used in 1 case. FNA was done in 13 cases and was accurate in 7 out of 13 cases (53.8%). It was non-diagnostic in 2 cases and misidentified 4 out of 13 (30.7%) cases as papillary thyroid cancer with the correct diagnosis of colorectal metastases determined after thyroidectomy and pathological analysis.

Immunohistochemical staining can differentiate between metastatic colorectal carcinoma and primary thyroid cancer. The immune markers CK20, CDX2 and Villi protein (Villin) are positively expressed in colorectal adenocarcinoma with absence of thyroglobulin and calcitonin [30]. The thyroid specific biomarkers of TTF-1 and PAX8 are usually positively expressed in primary thyroid cancer [30] and were absent in our case. The cytopathological diagnosis of colorectal metastases in the thyroid based on the FNA specimen is certain as it was also confirmed by findings from the liver biopsy specimen.

Early-stage colorectal cancer is highly treatable with surgery and adjuvant chemotherapy and a 5-year survival rate around 90% [31]. Colorectal screening programmes are established in many countries to diagnose colorectal cancer early. A large scale multi-national review of more than 3 million patients found that countries with long-standing colorectal screening had the largest decreases in colorectal cancer mortality [32]. Unfortunately, our patient did not participate in a screening programme which may have enabled earlier diagnosis and curative treatment.

As our patient had Stage IVB Colorectal cancer with widespread metastases (Colon and Rectum, American Joint Committee on Cancer (AJCC) 8th Edition – cT4cN0pM1b), a curative management strategy was not possible, and the patient was treated with palliative chemotherapy and symptom alleviation. The prognosis for stage IV colorectal cancer is poor with 5-year survival of < 10% and median survival of 5 months without chemotherapy [33]. Combination chemotherapy can increase survival to 11 months, while effectively palliating symptoms [34]. The role of surgical intervention in stage IV colorectal cancer is mainly to prevent obstructive symptoms or treat perforation through palliative procedures such as a colonic stent or diverting stoma.

A meta-analysis has shown that surgical resection of the thyroid for metastatic non-thyroid malignancies is associated with improved survival duration compared with non-surgical management [35]. There is no significant survival difference between total thyroidectomy and partial thyroidectomy [35]. In our review, all cases underwent surgery with total thyroidectomy performed in 7 out of 14 (50%) cases, hemithyroidectomy in 6 out of 14 (42.8%) cases and sub-total thyroidectomy in 1 case. However, in all cases surgical resection of the primary tumour and lung metastases had already occurred so the survival benefit from thyroid surgery may not be seen in aggressive cancers that present with multiple, synchronous metastases. In cases involving widespread neoplastic dissemination such as our patient, the role of thyroid surgery is confined to the relief of localised, obstructive symptoms due to tracheal and esophageal compression.

## Conclusions

This case shows that the thyroid can be a site of metastatic disease and a rare presentation of metastatic colorectal cancer. It is also the first reported case to our knowledge of metastatic colorectal cancer in the thyroid with concurrent autoimmune hyperthyroidism.

It demonstrates the utility of FNA biopsy of the thyroid, which can diagnose colorectal metastases in expert hands and can be performed easily compared to other histology sampling techniques. It also encourages pathologists to be vigilant to the possibility of unexpected metastatic tumours in specimens and to routinely use specific immunohistochemical markers for accurate diagnosis, in case the origin of malignancy is unclear.

Thyroid surgery can potentially improve survival in selected cases, but its primary role is in the alleviation of obstructive symptoms in patients with colorectal thyroid metastases. Encouraging participation in colorectal screening programmes is likely to have the biggest impact in improving survival.

## Data Availability

The data used in this publication is stored in the electronic medical record of the patient.

## Abbreviations

PET: Positron Emission Tomography
CT: Computed Tomography
FNA: Fine Needle Aspiration
CK20: Cytokeratin 20
CDX2: Caudal-related homeobox transcription factor 2
CK7: Cytokeratin 7
WT1: Wilms Tumour 1
P63: Tumour protein 63
TTF-1: Thyroid Transcription Factor 1
Pax8: Paired Box Protein
Villin: Villi protein
